# A Cross-Cultural Study of Health Interests and Pleasure by Consumers in 10 Countries

**DOI:** 10.3390/foods14213615

**Published:** 2025-10-23

**Authors:** Chunxiao Pan, Edgar Chambers, Jeehyun Lee

**Affiliations:** 1 Department of Food Science and Nutrition and Kimchi Research Institute, College of Human Ecology, Pusan National University, Busan 46241, Republic of Korea; panchunxiao@pusan.ac.kr; 2 Center for Sensory Analysis and Consumer Behavior, Kansas State University, Manhattan, KS 66506, USA

**Keywords:** health, taste, attitude, scale, general health interest, cross-culture, liking, acceptability

## Abstract

Understanding how individuals balance health and pleasure in food choices is important for promoting healthier diets. This study examined 6300 adults across ten countries (630 per country) using the General Health Interest and Pleasure subscales of the Health and Taste Attitude Scales. Participants were grouped into four categories—HH-HP (High Health, High Pleasure), HH-LP (High Health, Low Pleasure), LH-HP (Low Health, High Pleasure), and LH-LP (Low Health, Low Pleasure)—based on their scores. Clear cross-national differences were observed. Respondents in Peru and China prioritized both health and pleasure, while those in Mexico and Russia scored higher on pleasure but lower on health. A polarized pattern was found in Japan, and a more balanced distribution appeared in Thailand and Spain. Australia, the United Kingdom, and the United States showed generally lower scores for both dimensions. Females tended to report higher health interest and greater pleasure in eating than males. Older age and higher education were also associated with stronger interest in health and food enjoyment. These results emphasize the importance of considering cultural and demographic variations when designing strategies to encourage healthy eating, and they support the cross-cultural validity of the Health and Taste Attitude Scales.

## 1. Introduction

As diet-related chronic diseases continue to increase worldwide, they have become a major public health concern. According to the Global Burden of Disease (GBD) study and other large-scale epidemiological reports, unhealthy eating habits are a primary cause of non-communicable diseases, including obesity, diabetes, and cardiovascular conditions [1,2]. Consequently, international health organizations such as the World Health Organization (WHO) emphasize healthy eating as a central strategy for the prevention and management of these conditions [3,4]. In response to these challenges, researchers have emphasized the need for effective, personalized, and culturally appropriate behavioral strategies that consider the multiple motivations behind food choices [5,6].

Studies show that food choices often are influenced primarily by two key factors: health and pleasure [7,8,9]. Individuals with strong health motivations are more likely to consume nutrient-dense foods, such as fruits and vegetables [10], whereas those who prioritize pleasure often exhibit dietary patterns linked to higher intake of energy-dense, nutrient-poor foods high in fat, sugar, and salt [11]. Although these motivations are often viewed as conflicting, some studies indicate that these two concepts can be harmonized. Landry et al. (2018) suggested that many individuals perceive dietary pleasure in a way that is compatible with healthy eating [8]. Similarly, H. T. Luomala et al. (2006) argued that a balanced healthy lifestyle requires occasional indulgence [12]. We believe it is important to note that pleasure is an important part of eating and ‘feeding the mind’ can be a key part of emotional health.

Cultural background also shapes how people understand health and pleasure in food, influencing both attitudes toward food and its role in daily life [13,14]. Achieving a balance between health and pleasure in dietary decisions is a significant challenge that requires understanding cultural differences in consumer attitudes. For instance, American consumers often associate unhealthy foods with better pleasure and greater enjoyment [15]. In contrast, French consumers tend to have the opposite belief, as demonstrated by Werle et al. (2013) [16], suggesting that the health–pleasure divide may be culturally conditioned [8]. Previous cross-national studies show some patterns: Americans emphasize health, while French and Belgian consumers prioritize pleasure; Japanese consumers tend to value both equally, and in Russia, pleasure is the primary criterion for food choice, while health is less prioritized [17]. Chinese consumers consider both pleasure and health in their food choices [18,19,20]. It must be noted that those studies used various measures to determine the emphasis that consumers place on health and pleasure.

To assess consumer attitudes related to these two dimensions, the Health and Taste Attitude Scales (HTAS) provides a tool to measure attitudes toward the healthiness and pleasure of foods, with validations across multiple countries and regions [21], including Finland, the Netherlands, the United Kingdom [22], Taiwan [23], Serbia [24], Italy [9], and South Africa [25].

Considering the increasing integration of global food markets, comprehending cross-cultural attitudes becomes increasingly important [26,27,28]. However, it is critical to employ the same research tool to make comparisons. This study uses the General Health Interest (GHI) and Pleasure subscales of the HTAS to investigate how attitudes toward health and pleasure in food differ across ten countries: Australia, China, Japan, Mexico, Peru, Russia, Spain, Thailand, the United Kingdom, and the United States. The objectives of the research are to (a) determine if the subscales are valid in the various countries, and (b) compare whether countries are more health or pleasure-focused. Additionally, this research explores how demographic factors, such as sex, age, and education, influence these attitudes.

## 2. Materials and Methods

### 2.1. Participants

This study forms a section on health and pleasure related to food which was part of a broader international study with a total of 6300 adult consumers who were surveyed across ten countries: Australia, China, Japan, Mexico, Peru, Russia, Spain, Thailand, the United Kingdom (UK), and the United States (US) (Table 1). In each country, 630 respondents were drawn from existing Qualtrics panels using quota sampling. Quotas ensured equal representation by sex (50% male, 50% female) and by age group (18–34 years, 35–54 years, and ≥55 years, each comprising roughly one third of the sample). Participants did not receive direct financial compensation; instead, they took part through the Qualtrics incentive program [29]. Additional demographic data collected included the highest education level and household composition (number of adults and children). The study received approval from the Kansas State University Institutional Review Board under protocol #8492.

### 2.2. Questionnaire

In this study, two specific HTAS subscales, GHI and Pleasure, are used (Table 2). Each subscale contains an equal number of positively and negatively phrased statements, with responses recorded on a seven-point Likert scale ranging from “strongly disagree” to “strongly agree.” Consumers were asked to comment on their habits, not specific foods.

The questionnaire, including demographic and other questions, was translated into Japanese, simplified Mandarin, Russian, Spanish, and Thai for deployment in the respective countries. A variation of the TRAPD (Translation, Review, Adjudication, Pretesting, and Documentation) methodology, as detailed by Seninde and Chambers (2020) and rooted in the works of Curtarelli and van Houten (2018) and Harkness (2003) [30,31,32], guided the translation process. Initially, a researcher in the relevant field, who was a native speaker fluent in English, translated the English questionnaire into the target language. Subsequently, a second researcher in the same area, also a native speaker, independently translated the document back into English. Both translators then collaborated, either in person or virtually, to reconcile the translations and ensure conceptual equivalence. In rare instances of disagreement, a third-party adjudicator was prepared to intervene; however, consensus was achieved in all cases without requiring external mediation. This process aligns with established practices in multilingual survey studies [29,33,34]. Each translated version underwent a “soft launch,” involving 50 participants from the target country, to assess clarity, completion feasibility within the designated time frame, and data quality. Feedback from screening and attention-check items confirmed that participants comprehended the questions, and the collected data were complete, reasonable, and valid. Following this validation phase, the translations were approved for full-scale use in the study.

### 2.3. Data Analysis

After re-coding (reverse scoring) the negatively worded items, the construct validity of the HTAS was assessed by analyzing the internal consistency reliability of its scales for each of the countries. Cronbach’s alpha was used to determine the reliability of each subscale. Pearson’s correlation analysis was conducted to assess the relationships between the subscales. Mean scores for each item were calculated to compare differences in GHI and Pleasure across countries.

Participants were divided into four groups based on median values of GHI and Pleasure: HH-HP (High Health, High Pleasure), HH-LP (High Health, Low Pleasure), LH-HP (Low Health, High Pleasure), and LH-LP (Low Health, Low Pleasure). The distribution of scores within these groups was compared across different countries. Analysis of variance (ANOVA) was performed to detect significant differences in GHI and Pleasure scores based on demographic factors such as country, sex, age, education level, and household size. When significant differences were found, post hoc analysis was conducted using the least significant difference (LSD) method. Data analysis was performed using SAS version 9.4 (SAS Institute Inc., Cary, NC, USA) and RStudio version 1.3.1093 (RStudio Team, 2020).

## 3. Results

### 3.1. Reliability and Correlation of the GHI and Pleasure Subscales

Cronbach’s α assessed internal consistency for each subscale across the ten countries. The GHI subscale showed strong reliability (α = 0.77 to 0.88). Removing item 13 (“I finish my meal even when I do not like the taste of the food”) raised the Pleasure subscale’s α above 0.53, meeting the threshold for moderate reliability [35]. GHI and Pleasure scores were positively correlated (r = 0.25, *p* < 0.001), indicating that greater health interest is linked with higher eating pleasure. Most item–pair correlations were moderate and positive, whereas item 13 had weak associations with other items (median r ≈ 0.06), supporting its exclusion from further analysis [36].

### 3.2. Cross-Cultural Differences in Health and Pleasure Attitudes

**Figure 1:**
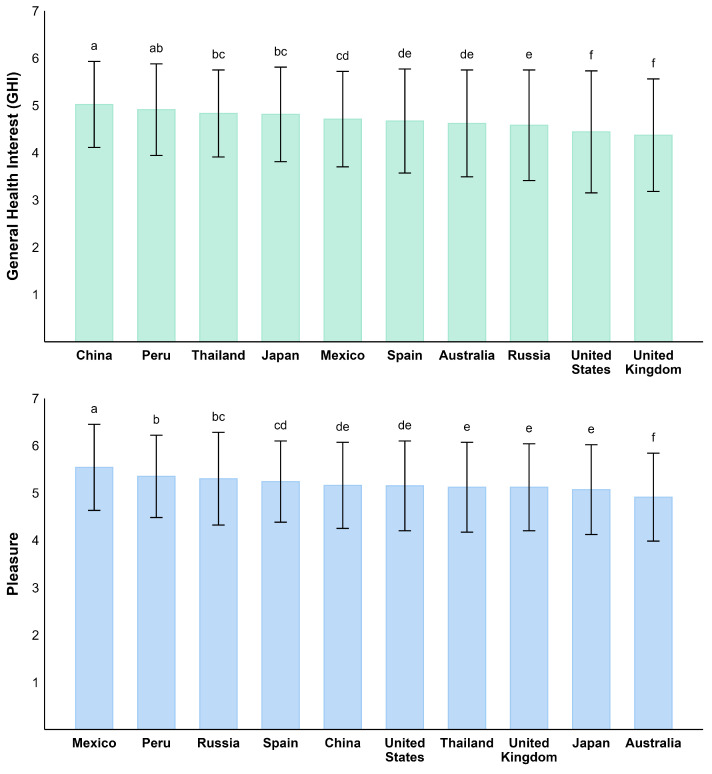
Cross-cultural comparison of General Health Interest (GHI) and Pleasure by country, high to low. Different letters indicate statistically significant differences between countries based on pairwise comparisons.

One-way ANOVA revealed significant country effects on both GHI and Pleasure scores (*p* < 0.001; Figure 1). Across all nations, mean Pleasure scores (4.91–5.54) were consistently higher than mean GHI scores (4.37–5.02). For GHI, China had the highest mean score, followed by Peru and Thailand, indicating a stronger focus on health-related food choices in these countries. In contrast, the US and UK exhibited the lowest GHI scores, suggesting a comparatively lower interest in health-conscious eating. For pleasure, Mexico and Peru showed the highest pleasure of food, while Australia had the lowest scores.

**Figure 2:**
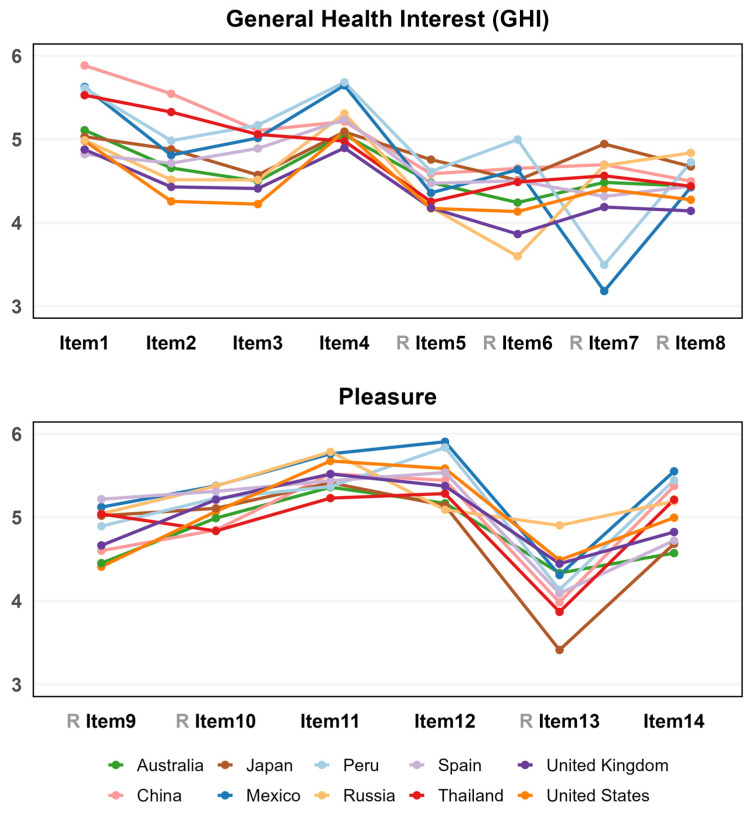
Average scores of the 14 items of the questionnaire across 10 countries.

Figure 2 shows the cross-country differences in scores across individual items of the GHI and Pleasure scales. After recoding the negatively worded items, all scores could be interpreted in the same direction.

On the GHI scale, items such as “I am very particular about the healthiness of food” (Item 1) and “It is important for me that my daily diet contains a lot of vitamins and minerals” (Item 4) scored higher across most countries, indicating a broad concern for healthy eating. Mexico and Peru scored higher on micronutrient-related items, such as “It is important for me that my daily diet contains a lot of vitamins and minerals” (Item 4) and “I do not avoid foods, even if they may raise my cholesterol” (Item 6), but had lower scores on item 7 “The healthiness of food has little impact on my food choices”. In contrast, Japan scored higher on item 7.

On the Pleasure scale, items such as “I need to eat delicious food on weekdays as well as weekends” (Item 11) and “When I eat, I concentrate on enjoying the taste of food” (Item 12) consistently scored high across countries. However, scores for item 13, “I finish my meal even when I do not like the taste of food” were generally low across all countries. Japanese participants scored even lower, showing a stronger reluctance, while Russian participants scored slightly higher, indicating a comparatively greater willingness to complete their meals despite disliking the taste. This statement does not necessarily support the pleasure construct and may have some relation to cultural norms related to income and food access.

Across both the GHI and Pleasure scales, the scores for negatively worded items were generally lower than those for positively worded items, suggesting potential cognitive differences in how participants approached reverse-coded questions.

**Figure 3:**
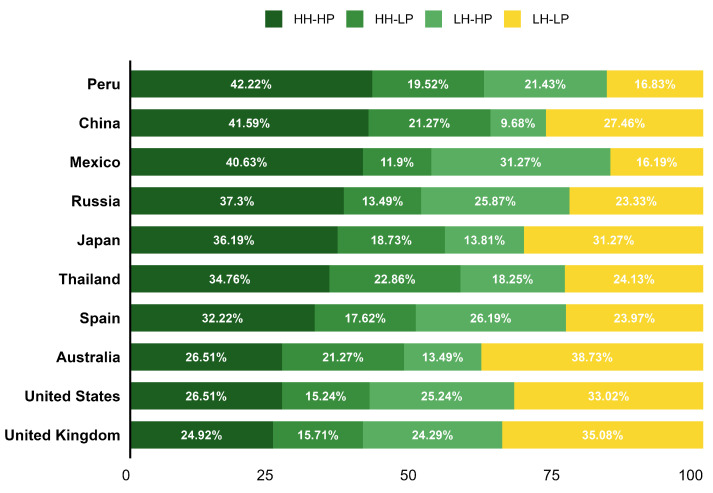
Distribution of Health and Pleasure groups across 10 countries. Distribution of respondents across 10 countries based on “general health interest” and “pleasure” scores from the Health and Taste Attitude Scale, categorized as HH-HP (High Health, High Pleasure), HH-LP (High Health, Low Pleasure), LH-HP (Low Health, High Pleasure), and LH-LP (Low Health, Low Pleasure).

Many previous studies have suggested that health and pleasure are often perceived as opposites, and their relationship varies across cultures [7,8,9,10,25,37]. To investigate whether this opposition exists across different countries, participants were classified into four HTAS groups based on their Health (GHI) and pleasure preferences (excluding item 13), following the method described by H. Luomala et al. (2015) [37]. HH-HP represents high health and pleasure, HH-LP represents high health and pleasure, LH-HP represents low health and high pleasure, and LH-LP represents low health and pleasure (Figure 3).

The chi-square test (χ^2^ = 359.68, *p* < 0.0001) showed significant differences in the distribution of health and pleasure preferences across countries. On average, the combined percentage of participants in the HH-HP and LH-LP groups across all countries was 61%, indicating that most participants held consistent views on both health and pleasure.

In China and Peru, the proportion of participants in the HH-HP group was relatively high, reflecting a strong dual focus on both health and pleasure.

In Mexico and Russia, higher proportions of both the HH-HP and LH-HP groups were observed, suggesting that consumers in these countries tend to prioritize pleasure, with health concerns varying between individuals.

In contrast, Japan had nearly equal proportions of participants in the HH-HP and LH-LP groups, reflecting a polarization in attitudes toward health and pleasure. Some participants valued a balance between health and pleasure, while others placed less emphasis on both aspects.

In Thailand and Spain, the distribution across the four HTAS groups was relatively balanced, indicating that participants in these countries showed a more diverse range of attitudes toward health and pleasure.

Australia, the UK, and the US saw the LH-LP group have the highest proportion, which suggests that most participants were less concerned with both health and pleasure as compared to other countries. It is also possible that English speakers are more comfortable using lower ranges on the scales.

### 3.3. Demographic Differences in Health and Pleasure Attitudes

**Figure 4:**
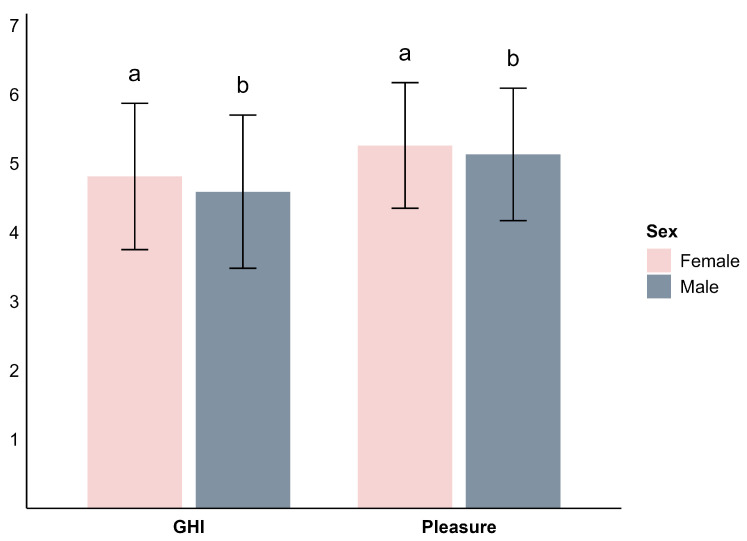
Comparison of General Health Interest (GHI) and Pleasure by sex. Different letters indicate statistically significant differences between countries based on pairwise comparisons.

The analysis revealed significant sex-based differences in the mean scores on HTAS (Figure 4). Female respondents reported a mean GHI score of 4.8 ± 1.1, which was significantly higher than the male score of 4.6 ± 1.1 (F = 67.5, *p* < 0.001). Similarly, females reported a higher Pleasure score (5.3 ± 0.9) compared to males (5.1 ± 1.0), with an F value of 33.1 (*p* < 0.001). These findings align with prior studies [9,22,23,38], which consistently suggest that females are more health-conscious and tend to derive greater pleasure from food compared to males.

A cross-cultural comparison of GHI and Pleasure across age groups showed significant differences (Table 3). Adults aged 55 and above had the highest GHI scores, significantly exceeding those of the 35–54 and 18–34 age groups. For Pleasure, the 35–54 age group had the highest scores, while no significant difference was found between the 18–34 and 55 years old or older groups in Pleasure scores. The results validate previous findings that motivation for healthy eating increases with age [21,38].

Educational attainment was significantly associated with both GHI and Pleasure scores (Table 4). Participants with a college or university degree reported the highest scores on both scales, followed by those with a high school education, while the lowest scores were observed among individuals with primary education or less. This gradient aligns with evidence linking higher educational levels to improved diet quality [39], which may shape attitudes toward both health-related and hedonic aspects of food.

The number of adults in the household was related to both GHI and Pleasure scores (Table 5). GHI scores were generally higher in larger households, with the lowest values observed among single-adult households. Pleasure scores showed a clearer increasing trend, reaching the highest levels in six-adult households.

In contrast, the number of children in the household was not related to GHI, as scores remained consistent across groups and no significant differences were observed (Table 6). However, Pleasure scores varied with child number. Households with one to three children reported higher Pleasure scores than those with no children or four or more children.

## 4. Discussion

### 4.1. Interpretation of Cross-Cultural Differences in Health and Pleasure Attitudes

The results of this study indicated that the relationship between health and pleasure varied significantly across different cultures. Although health and pleasure are often viewed as opposing motivations, most participants exhibited consistent orientations toward both, with high or low scores tending to co-occur. This suggests that, in many cultural contexts, health and enjoyment may not be mutually exclusive but instead reflect a shared evaluative stance on food. However, this did vary by individual consumers. Approximately 30–40% of consumers in each country showed the opposite trend with consumers scoring health high and pleasure low and vice versa. Not all consumers viewed these similarly. Hofstede [40] proposed a widely accepted theory that cultural differences are related to six main factors: (a) power distance (related to human inequality), (b) uncertainty avoidance (societal stress in dealing with uncertainty), (c) individualism (vs collectivism), (d) masculinity versus femininity (now often known as motivation to achievement and success), (e) long-term versus short-term orientation (present/past vs. future), and (f) indulgence (versus restraint). That author notes a correlation with a country’s wealth for both dimensions a and c. Thus, wealth must always be considered when trying to understand cultural dimensions. A website [41] provides scores for each of those dimensions for all the countries in our study for us to use to help determine if those cultural difference categories appear to explain these data.

In China and Peru, consumers place equal emphasis on both health and pleasure in their food choices, a reflection of traditional cultural beliefs and dietary practices. In China, the concept of “medicine and food sharing the same source” is deeply ingrained, with food viewed not only as a nutritional source but also as a tool for prevention and healing [42]. Similarly, Peruvian food culture emphasizes the use of diverse, natural ingredients such as fruits and vegetables, supported by rich agricultural resources. The increasing consumption of environmentally sustainable proteins and less processed carbohydrates further highlights a focus on healthy eating [43]. Interestingly, these two countries are not necessarily similar in relation to the dimensions proposed by [40]. Although both countries score high for power differentiation (e.g., wealth) (website), but China scores much lower for uncertainty avoidance and much higher for long-term focus than Peru. Both score in the lower half of the indulgence scale. This does not seem to necessarily relate to patterns of high food pleasure and health, where one might assume there is more interest in indulgence and long-term thinking.

In contrast, Mexico and Russia exhibited a stronger orientation toward pleasure over health. Mexican cuisine is known for its complex flavors and cultural significance, which contribute to a heightened focus on sensory enjoyment [44]. In Russia, dietary patterns are centered on meat consumption, with food pleasure often prioritized over health concerns [17,45]. In terms of the cultural dimensions’ scales noted previously, Mexico and Russia both score high for power differential and uncertainty avoidance, but they differ in motivation for success and achievement (Mexico higher), long-term orientation (Russia higher), and indulgence (Mexico much higher than Russia). These findings suggest that the cultural similarities in pleasure and health related to food may be more complex and nuanced than an overall theory of cultural differences can explain.

Japan displayed a polarized distribution of attitudes. This reflects the diversity within its food culture, where both traditional health-oriented values and modern convenience-based preferences coexist [46]. It is interesting to note that on Hofstede’s cultural scales, Japan was not similar to any of the other countries we studied.

In both Thailand and Spain, participants were evenly distributed across the four HTAS groups, reflecting a broad range of perspectives on health and pleasure. In Thailand, studies link health risk perceptions with happiness, having a sufficient intake of fruits and vegetables along with positive attitudes toward health and food safety is associated with higher levels of happiness [47]. Similarly, research in Spain shows that consumer opinions on functional foods and the impact of a healthy lifestyle on eating habits demonstrate a balanced approach to health and pleasure [48]. For Hofstede’s cultural scales, Spain and Thailand exhibited some similarities in most categories but differed tremendously in terms of individualism (Spain was higher).

In the US and the UK, although over 50% of participants were consistent in terms of health and pleasure, the majority of their scores fell in the LH-LP group. This suggests that health and pleasure are not as important in these countries, which may be attributed to a utilitarian approach to food in these cultures. For example, American families tend to place more importance on the nutritional function of food than on its pleasure value [49,50], contributing to a view of food more as a risk than a source of enjoyment [51]. Hofstede’s scales show similarities among UK, US, and Australian consumers similar to this research. It must be noted that these three countries also have among the highest wealth of the countries studied.

### 4.2. Influence of Demographic Factors on GHI and Pleasure

Sex differences in health and pleasure attitudes, as measured by the HTAS, were consistently observed, with females reporting higher scores on both scales than males [9,22,38]. These findings are consistent with prior research showing that women are more likely to avoid high-fat foods, consume more fruits and fiber, and place greater importance on healthy eating [52]. Women also tend to possess higher nutritional knowledge and are more likely to seek dietary counseling [53], further supporting the observed differences in health- and pleasure-related attitudes.

Age is also an important factor in health and pleasure scores. Studies have shown that participants aged 54 years and older exhibit the highest health interest scores, while those aged 35–54 exhibit the highest pleasure scores. Consumer attitudes toward food attributes have changed over time, with responses related to texture and flavor becoming less prominent, while health-related responses have become more frequent with age [54,55]. It must be emphasized, however, that people still only choose foods they like to eat and avoid those that they dislike.

Education also plays a critical role in shaping attitudes toward health and pleasure. Highly educated individuals (college and above) showed higher scores in both health interest and pleasure. Individuals with higher education tend to consume more fruits and vegetables and less sugar- and fat-rich foods compared to those with only primary education [56]. Education also affects how individuals balance pleasure and health in their food choices. Higher levels of education are associated with a greater ability to balance food pleasure and health goals [57].

Household composition also contributed to observed differences. The number of adults in a household was positively related to both GHI and Pleasure scores, with larger households reporting higher levels on both dimensions. This may reflect the influence of shared food environments and increased social interaction [58]. In contrast, the number of children had minimal impact on GHI but was associated with variation in Pleasure scores. Households with one to three children reported higher Pleasure scores than those with no children or four or more, suggesting that a moderate family size may support more enjoyable eating environments [59,60].

### 4.3. Applicability and Limitations of the Health and Taste Attitude Subscales

The HTAS has been shown to be a valid instrument for assessing health and pleasure attitudes across different cultures. However, scores of respondents were generally lower for statements that were negatively framed than for statements that were positively framed, which suggests cognitive differences in their approach to reverse-coded questions. Reverse-coded questions are typically designed to impose a higher cognitive load on respondents [61], and scores may be influenced by respondents’ inattention and confusion [62]. Moreover, the factors affecting the score of item 13 (“I finish my meal even when I do not like the taste of a food”) are multifaceted, encompassing various social, economic, and cultural factors, rather than just pleasure aspects [63]. Therefore, it is important to consider revising or redesigning the scale in future research to improve its applicability.

### 4.4. Limitations of the Study

Although this study included participants from various countries, there are limitations regarding the representativeness of the sample. The sample in developing nations may not adequately capture the perspectives of consumers in low-income or remote regions. Additionally, the use of an online questionnaire may have excluded non-internet users, which could have further restricted the study’s generalizability. In addition, we studied only a portion of the potential demographic or psychographic variables that could impact responses to this study.

The scales themselves could have impacted the results. For example, the decision not to use the full HTAS scale may have limited the study’s ability to fully capture participants’ attitudes toward health and pleasure. However, various methods for testing eating motivations, both with surveys and interviews, have shown consistency among findings that health and pleasure can be measured predictably for meals and snacking occasions [64]. The imbalance between positive and negative items on the pleasure scale, especially after the removal of item 13, could have affected response consistency [65]. It also must be noted that the HTAS measures potential attitudes, not behaviors. It is well known that attitudes may influence, but do not directly translate to behaviors [66]. Various authors [67,68] suggested that using commonly used theories, such as the Theory of Planned behavior, for predicting food behavior modifications provides some predictability (14–24%), but the applicability and predictability depends strongly on the specific behaviors [66,67]. Other more general theories, such as Hofstede’s Cultural Dimensions [40] may not provide the specificity needed to understand food choices.

## 5. Conclusions

This cross-cultural study, which uses the same research tool to understand health and pleasure aspects of food, highlights the complex relationship between health and pleasure preferences, demonstrating that cultural and demographic factors significantly shape consumer attitudes. However, the results do not fit neatly into specific theories of cultural differences. These findings have important implications for public health initiatives, suggesting that culturally sensitive strategies that consider both pleasure and health are necessary to promote healthier eating habits. It is clear that many people consider both health and pleasure when eating, which means that foods that are both pleasurable and promote good health must be available to those populations. Professionals must emphasize the need for a healthy diet, rather than the consumption of specific foods for physical health. We believe it is important to feed both mind and body, which recognizes that pleasurable eating and eating experiences are important aspects of long-term healthy eating behaviors.

## Figures and Tables

**Table 1:** Demographic characteristics of participants in the 10 countries are shown in percentages (n = 6300).

	Australia	China	Japan	Mexico	Peru	Russia	Spain	Thailand	UK	US
Sex										
Female	50.0	50.0	50.0	50.00	50.0	50.0	50.0	50.0	49.8	49.8
Male	50.0	50.0	50.0	50.00	50.0	50.0	50.0	50.0	50.2	50.2
Age										
18–34 years old	33.3	33.3	33.3	33.3	33.3	33.3	33.3	33.3	33.3	33.2
35–54 years old	33.3	33.3	33.3	33.3	33.3	33.3	33.3	33.3	33.3	33.5
55 years old or older	33.3	33.3	33.3	33.3	33.3	33.3	33.3	33.3	33.3	33.3
Education (Highest Degree)										
Primary school or less	3.7	0.6	2.1	0.32	0.3	0.8	4.6	1.8	12.9	1.3
High School	36.2	20.6	33.2	4.13	9.4	8.1	42.4	12.5	26.0	45.4
College/Univ. grad.	60.2	78.7	64.8	95.6	90.3	91.1	53.0	85.7	61.1	53.3
Number of Adult(s) in Household										
1	21.0	2.2	17.3	0.5	0.8	11.8	0.2	11.4	19.5	21.0
2	54.9	20.5	35.7	5.7	3.3	51.9	9.8	18.9	55.9	54.9
3	14.6	51.8	24.8	40.2	29.4	21.9	50.5	22.4	15.1	14.6
4	6.2	12.5	15.6	22.1	22.2	10.0	23.0	28.1	6.8	6.2
5	3.3	13.0	6.7	17.6	23.7	4.4	13.8	19.2	2.7	3.3
6	0.0	0.0	0.0	14.0	20.6	0.0	2.7	0.0	0.0	0.0
Number of Child(ren) in Household										
0	66.5	42.5	75.1	37.8	39.4	51.8	58.4	47.8	63.7	66.5
1	17.3	50.3	16.8	28.4	27.0	26.8	25.9	30.2	16.8	17.3
2	11.9	6.8	6.4	25.6	23.0	17.5	13.5	17.0	14.0	11.9
3	3.7	0.2	1.4	6.0	8.3	3.5	2.2	2.7	4.1	3.7
4 or more	0.6	0.2	0.3	2.2	2.4	0.5	0.0	2.4	1.4	0.6

**Table 2:** Subscales of the Health and Taste Attitude Scales (HTAS).

General Health Interest		
1		I am very particular about the healthiness of food
2		I always follow a healthy and balanced diet
3		It is important for me that my diet is low in fat
4		It is important for me that my daily diet contains a lot of vitamins and minerals
5	R *	I eat what I like, and I do not worry much about the healthiness of food
6	R	I do not avoid foods, even if they may raise my cholesterol
7	R	The healthiness of food has little impact on my food choices
8	R	The healthiness of snacks makes no difference to me
Pleasure		
9	R	I do not believe that food should always be a source of pleasure
10	R	The appearance of food makes no difference to me
11		I need to eat delicious food on weekdays as well as weekends
12		When I eat, I concentrate on enjoying the taste of food
13	R	I finish my meal even when I do not like the taste of a food
14		An essential part of my weekend is eating delicious food

* Negative statements are marked with an “R” after the statement number.

**Table 3:** Comparison of General Health Interest (GHI) and Pleasure by age.

	Age	Mean ± std	Grouping	F Value	Pr (>F)
General Health Interest	18–34 years old	4.50 ± 1.07	c	62.8	<0.001
	35–54 years old	4.71 ± 1.10	b		
	55 years old or older	4.88 ± 1.08	a		
Pleasure	18–34 years old	5.18 ± 0.92	b	12.4	<0.001
	35–54 years old	5.27 ± 0.91	a		
	55 years old or older	5.13 ± 0.98	b		

**Table 4:** Comparison of General Health Interest (GHI) and Pleasure by education.

	Education (Highest Degree Awarded)	Mean ± std	Grouping	F Value	Pr (>F)
General Health Interest	College or University graduate	4.77 ± 1.05	a	36.36	<0.001
	High School	4.51 ± 1.17	b		
	Primary school or less	4.46 ± 1.21	b		
Pleasure	College or University graduate	5.24 ± 0.94	a	19.88	<0.001
	High School	5.10 ± 0.91	b		
	Primary school or less	4.93 ± 0.95	c		

**Table 5:** Comparison of General Health Interest (GHI) and Pleasure by number of adult(s) in household.

	Number of Adult(s) in Household	Mean ± std	Grouping	F Value	Pr (>F)
General Health Interest	1	4.52 ± 1.23	c	8.12	<0.001
	2	4.65 ± 1.10	b		
	3	4.75 ± 1.08	ab		
	4	4.70 ± 1.05	b		
	5	4.85 ± 1.01	a		
	6	4.75 ± 1.03	ab		
Pleasure	1	5.01 ± 0.97	e	15.8	<0.001
	2	5.13 ± 0.94	d		
	3	5.24 ± 0.93	c		
	4	5.22 ± 0.93	cd		
	5	5.35 ± 0.90	b		
	6	5.45 ± 0.86	a		

**Table 6:** Comparison of General Health Interest (GHI) and Pleasure by number of child(ren) in household.

	Number of Child(ren) in Household	Mean ± std	Grouping	F Value	Pr (>F)
General Health Interest	0	4.67 ± 1.13	–	1.96	0.098
	1	4.76 ± 1.03	–		
	2	4.70 ± 1.05	–		
	3	4.69 ± 1.11	–		
	4 or more	4.67 ± 1.17	–		
Pleasure	0	5.10 ± 0.96	b	21.12	<0.001
	1	5.32 ± 0.89	a		
	2	5.33 ± 0.91	a		
	3	5.30 ± 0.88	a		
	4 or more	5.06 ± 1.00	b		

## Data Availability

The original contributions presented in this study are included in the article. Further inquiries can be directed to the corresponding author.
